# Electrochemical Skin Conductance by Sudoscan in Non-Dialysis Chronic Kidney Disease Patients

**DOI:** 10.3390/jcm13010187

**Published:** 2023-12-28

**Authors:** Liang-Te Chiu, Yu-Li Lin, Chih-Hsien Wang, Chii-Min Hwu, Hung-Hsiang Liou, Bang-Gee Hsu

**Affiliations:** 1 Division of Nephrology, Department of Internal Medicine, Dalin Tzu Chi Hospital, Buddhist Tzu Chi Medical Foundation, Chiayi 62247, Taiwan; carol75820@gmail.com; 2 Division of Nephrology, Hualien Tzu Chi Hospital, Buddhist Tzu Chi Medical Foundation, Hualien 97004, Taiwan; nomo8931126@gmail.com (Y.-L.L.); wangch33@gmail.com (C.-H.W.); 3 School of Medicine, Tzu Chi University, Hualien 97004, Taiwan; 4 Section of Endocrinology and Metabolism, Department of Medicine, Taipei Veterans General Hospital, Taipei 11217, Taiwan; chhwu@vghtpe.gov.tw; 5 Division of Nephrology, Department of Medicine, Hsin-Jen Hospital, New Taipei City 242009, Taiwan

**Keywords:** chronic kidney disease, diabetes, electrochemical skin conductance, neuropathy, Sudoscan

## Abstract

Background. Peripheral neuropathy is prevalent among patients with chronic kidney disease (CKD). Sudoscan non-invasively detects polyneuropathy by measuring electrochemical skin conductance (ESC). We conducted a study on sudomotor function in CKD patients across various stages based on their estimated glomerular filtration rate (eGFR). Methods. In this cross-sectional study of 700 CKD patients, all underwent Sudoscan. Pathological ESC was defined as hands < 40 μS or feet < 50 μS. Clinical neuropathy scores including Michigan Neuropathy Screening Instrument (MNSI) and Douleur Neuropathique en 4 questionnaire (DN4) were obtained. Results. Among participants, 344 had diabetes and 356 did not. Hands and feet ESC decreased with CKD progression (median (IQR) in stage 1–2, 3, 4–5: 54.0 (39.0–68.0), 45.5 (30.0–63.0), 41.8 (26.5–60.5), *p* trend < 0.001; 64.5 (53.5–74.0), 60.5 (43.0–72.5), 55.0 (39.0–69.8), *p* trend < 0.001). Pathological hands and feet ESC increased in later CKD stages (stage 1–2, 3, 4–5: 26.6%, 40.9%, 45.7%, *p* trend < 0.001; 21.7%, 34.0%, 40.6%, *p* trend < 0.001). Positive hands and feet ESC-eGFR correlation existed irrespective of diabetes. Diabetic patients had lower hands and feet ESC than non-diabetics as CKD progressed. However, multivariate regression found no significant ESC-eGFR association. Sudoscan correlated with clinical neuropathy scores. Conclusion. Pathological sudomotor function was common in non-dialysis CKD stages 4–5. Diabetic patients had worse function. Sudomotor dysfunction progressed with renal disease but eGFR was not an independent risk factor.

## 1. Introduction

Patients with advanced chronic kidney disease (CKD) frequently suffer from peripheral neuropathy [1,2,3], which has been regarded as a key risk factor for foot ulceration and lower-extremity amputation [4]. The prevalence rate of peripheral neuropathy is around 60% in pre-dialysis CKD patients and can even reach 100% in dialysis patients [1,2,5,6]. This neuropathy commonly presents as a distal symmetrical process with sensorimotor involvement [1,2]. Furthermore, it is clinically indistinguishable from other neuropathies, such as diabetic neuropathy [1]. In the past, it was believed that this neuropathy only developed when the estimated glomerular filtration rate (eGFR) fell below 12 mL/min/1.73 m^2^ [2,3]. However, growing evidence has revealed that peripheral neuropathy begins in earlier stages of CKD, highlighting the need for awareness and early intervention [7,8,9].

The diagnosis of peripheral neuropathy requires the recognition of both clinical symptoms and signs, as well as nerve conduction studies. Nevertheless, early detection of diabetic polyneuropathy can be challenging due to a lack of findings in nerve conduction studies, often necessitating invasive skin biopsy to assess intra-epidermal nerve fiber density [10,11]. Sudoscan (Impeto Medical, Paris, France) is a handy and non-invasive device designed to measure cutaneous sweat gland function, which reflects the activity of innervating small un-myelinated C fibers. Sudoscan’s can be used in a variety of procedures, such as clinical examinations for neuropathy, measurements of peripheral sensory perception, pain scores, and to diagnose diabetic peripheral neuropathy [12]. Sudoscan has been confirmed to have similar diagnostic accuracy for distal symmetric polyneuropathy as skin biopsy in patients with and without diabetes [10]. Currently, studies measuring sudomotor function have only focused on diabetic patients in earlier stages of CKD [13,14]. This study aims to evaluate sudomotor function in both patients with and without diabetes across various stages of CKD.

## 2. Materials and Methods

### 2.1. Patients

This cross-sectional study enrolled a total of 700 CKD patients from a single medical center in Eastern Taiwan (Hualien Tzu Chi Hospital) from July 2021 to November 2022. All participants were over 20 years old and were recruited from the Nephrology Outpatient Department. Exclusion criteria included active malignancy, recent stroke, acute infection, amputated limb(s), or bedridden status. All patients provided written informed consent, and the study received ethics approval from the Research Ethics Committee, Hualien Tzu Chi Hospital (IRB109-282-A), conducted in accordance with relevant guidelines and regulations.

### 2.2. Data Collection

We collected patients’ epidemiological and laboratory data through chart review and inquiry. The demographic data collected included age, gender, body mass index (BMI), CKD stage, and the presence of diabetes, hypertension, or glomerulonephritis.

### 2.3. Biochemical Investigations

Blood samples were collected after an overnight fast. Hemoglobin measurements were performed using the Sysmex SP-1000i (Sysmex America, Mundelein, IL, USA). Serum levels of albumin, blood urea nitrogen (BUN), creatinine, total cholesterol (TCH), calcium, and phosphorus, as well as urinary levels of protein and creatinine, were measured using an autoanalyzer (Siemens Advia 1800, Siemens Healthcare GmbH, Henkestr, Germany). Glycohemoglobin (HbA1c) measurements were based on high-performance liquid chromatography (ADAMS A1c HA-8180V, Arkray, Inc., Kyoto, Japan). Estimated GFR was calculated using the equation developed for the Modification of Diet in Renal Disease (MDRD) Study [15]. The urine total protein to creatinine ratio (UPCR) was calculated from spot urine, as the total protein in g/dL divided by creatinine in g/dL. Calcium and phosphorus measurements were only performed in 509 participants (stage 1–2: *n* = 66; stage 3: *n* = 239; stage 4–5: *n* = 204).

### 2.4. Sudomotor Function Assessment

Sudoscan is an FDA-approved device (Impeto Medical Device, EZS 01750010193, Paris, France) designed to measure sudomotor function [12]. It estimates sweat gland function through reverse iontophoresis and chronoamperometry, measuring the electrochemical skin conductance (ESC) (sweat chloride ion current). The device consists of a desktop computer and two sets of flat stainless steel electrodes, onto which the subject places their palms and feet. Low-voltage direct currents are then incrementally applied. The sweat chloride ion current generated in response to the incremental voltages, which reflects sudomotor function, is a result of the innervation of sweat glands by C fibers. ESC is measured in micro Siemens (μS) and represents the ratio of the currents generated and applied to the electrodes. During the non-invasive two-minute scan, each extremity is individually assessed, and the device calculates the asymmetry (%) between the left and right extremities. Sudoscan also incorporates built-in algorithms that integrate ESC with additional biometric data to provide a cardiac autonomic neuropathy (CAN) score [16] and a nephropathy score that estimates the current risks of CKD [14].

In diabetic patients, an ESC of less than 40 µS indicates severe impairment of sudomotor function and is associated with advanced peripheral neuropathy [16,17]. However, the optimal threshold for detecting peripheral neuropathy in the Chinese CKD population is currently undefined. Based on a previous study conducted in the dialysis population by Reach et al. [18], we defined a pathological ESC as either a hands ESC value < 40 μS or a feet ESC value < 50 μS in our CKD patients.

### 2.5. Clinical Neuropathy Scores

The Michigan Neuropathy Screening Instrument (MNSI) and Douleur Neuropathique en 4 questionnaire (DN4) scores were obtained in a subset of study subjects following Sudoscan. The MNSI consists of 15 yes or no questions on foot sensation (MNSI_Q) and a physical exam, including foot inspection, vibration assessment, ankle reflex test, and monofilament testing (MNSI_P) [19]. Although the MNSI score was initially designed for diagnosing diabetic neuropathy, it has been validated for diagnosing uremic neuropathy in patients undergoing dialysis [20]. The clinical exam was performed by a nurse trained in its administration. DN4 is comprised of 10 questions and was validated for the diagnosis of neuropathic pain [21].

### 2.6. Statistical Analyses

We used the SPSS 21.0 software (version 19.0; SPSS Inc., Chicago, IL, USA) for statistical analyses. The normality of continuous variables was determined using the Kolmogorov–Smirnov test. Normally distributed data were reported as the mean ± standard deviation, while non-normally distributed data were reported as medians and interquartile ranges. Categorical data were reported as frequencies and percentages. The linear trend of variables across different CKD stages was tested using either one-way analysis of variance (ANOVA) or the Cochran–Armitage test for trend.

Spearman’s correlation analysis was used to evaluate the correlation between the hands and feet ESC scores and relevant clinical variables. A multivariate linear regression model was constructed to determine the independent factors associated with pathological hands and feet ESC. Variables of clinical relevance, including age, gender, diabetes mellitus, hypertension, body mass index, and eGFR, were adopted as covariates in multivariate model 1. Model 2 further adjusted for variables related to CKD, such as hemoglobin, albumin, and UPCR. Spearman’s correlation analysis was performed for hands and feet ESC scores and clinical neuropathy scores. A two-sided *p* < 0.05 was considered statistically significant.

## 3. Results

### 3.1. Clinical and Biochemical Characteristics of the Study Population Stratified by CKD Stages

The clinical characteristics of the 700 study subjects who met the inclusion criteria are presented in Table 1. The median age was 67 (59–76) years, and 404 (57.7%) were male. Patients with diabetes mellitus, hypertension, or glomerulonephritis comprised 49.1%, 78.1%, and 33.7% of the study subjects, respectively. The median estimated glomerular filtration rate (eGFR) was 37.9 (21.5–54.6) mL/min/1.73 m^2^. The median UPCR was 0.40 (0.16–1.34) g/g. The median hands ESC was 46.5 (30.5–63.5) μS with a median asymmetry of 8 (3–15) %, while the median feet ESC was 60.0 (43.0–72.0) μS with a median asymmetry of 5 (2–10) %. Pathological hands and feet ESC were observed in 278 (39.7%) and 237 (33.9%) patients, respectively. The median CAN score was 33 (28–38), and the median Nephropathy score was 46 (34–58).

Table 1 depicts the clinical characteristics of subjects comparing the CKD stage 1–2 (*n* = 143), stage 3 (*n* = 303), and stage 4–5 (*n* = 254) subgroups. Patients with more advanced CKD tended to be older, have a lower BMI, and have a higher prevalence of hypertension. Patients with earlier CKD tended to have higher hemoglobin, serum calcium, and albumin levels. Patients with advanced CKD exhibited higher proteinuria and serum phosphorus levels, lower hands and feet ESC, and increased prevalence of pathological ESC and Sudoscan asymmetry in both hands and feet.

### 3.2. Correlation between Sudoscan Score and Clinical and Biochemical Characteristics

Table 2 presents the results of the Spearman correlation analysis, examining the association between the Sudoscan score and other study parameters in all CKD patients. In the unadjusted analysis, hands and feet ESC showed a significant positive correlation with hemoglobin, serum albumin levels, and eGFR. Conversely, a significant negative correlation was observed between hands and feet ESC and age, presence of diabetes mellitus, hypertension, serum BUN, creatinine, and UPCR. Additionally, a significant positive association was found between hands ESC and BMI and between feet ESC and total cholesterol.

### 3.3. The Association between Sudoscan Score and CKD, Stratified by DM Status

**Figure 1:**
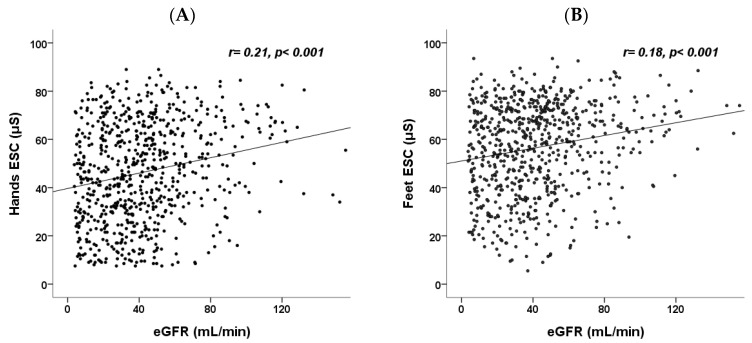

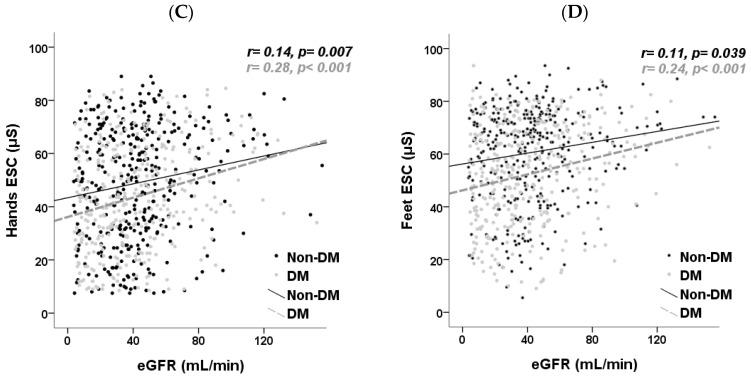
Scatter plots with linear regression depicting the relationship between (**A**) hands ESC and eGFR, (**B**) feet ESC and eGFR, (**C**) hands ESC and eGFR for non-DM and DM patients, and (**D**) feet ESC and eGFR for non-DM (*n* = 356) and DM (*n* = 344) patients. ESC, electrochemical skin conductance; eGFR, estimated glomerular filtration rate; DM, diabetes mellitus.

The relationship between hands ESC and feet ESC with eGFR was illustrated using scatter plots and analyzed using Spearman’s correlation coefficient in all CKD participants (Figure 1). Both hands ESC (r = 0.21, *p* < 0.001) and feet ESC (r = 0.18, *p* < 0.001) showed a significant correlation with eGFR. This correlation remained significant regardless of DM status (hands ESC in Non-DM: r = 0.14, *p* = 0.007; hands ESC in DM: r = 0.28, *p* < 0.001; feet ESC in Non-DM: r = 0.11, *p* = 0.039; feet ESC in DM: r = 0.24, *p* < 0.001; Figure 1).

**Figure 2:**
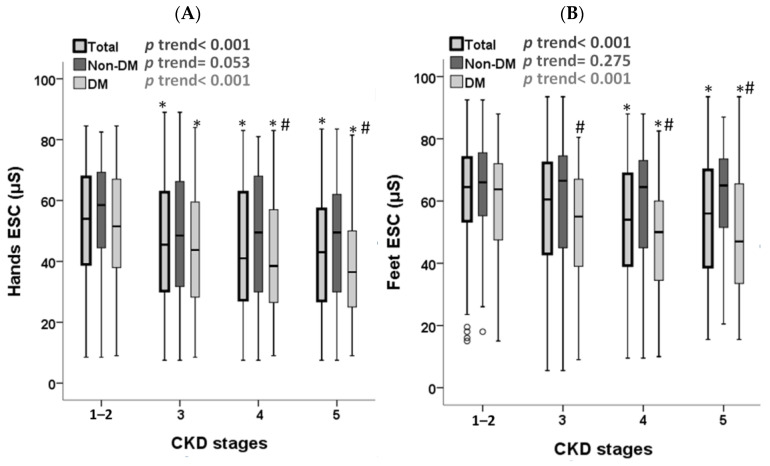
Box plots depicting the distributions of (**A**) hands ESC and (**B**) feet ESC in different CKD stages stratified by DM status. The box plots illustrate the 25th, 50th, and 75th percentiles as horizontal lines, while vertical lines extend to indicate the range between the 10th and 90th percentiles. * *p* < 0.05 vs. CKD stages 1–2; # *p* < 0.05 vs. the non-DM group. ESC, electrochemical skin conductance; CKD, chronic kidney disease; DM, diabetes mellitus.

The Sudoscan score in hands and feet demonstrated a significant decrease with the progression of CKD stages (hands ESC: *p* for trend < 0.001; feet ESC: *p* for trend < 0.001) as shown in Figure 2. This correlation persisted in CKD patients with diabetes (hands ESC: *p* for trend < 0.001; feet ESC: *p* for trend < 0.001), but not in patients without diabetes (hands ESC: *p* for trend = 0.053; feet ESC: *p* for trend = 0.275). Hands ESC significantly decreased in patients with CKD stages 3–5 compared to stages 1–2 in all CKD patients and CKD patients with diabetes, while feet ESC significantly decreased in CKD stages 4–5 compared to stages 1–2 in all CKD patients and CKD patients with diabetes. Additionally, in CKD stages 4–5, hands ESC was significantly lower in patients with diabetes compared to non-diabetics, and in CKD stages 3–5, feet ESC was significantly lower in patients with diabetes compared to non-diabetics.

In the univariate linear regression analysis, both hands and feet ESC showed significant associations with eGFR (β: 0.16 and 0.13 for hands ESC and feet ESC, respectively; Table 3). These associations remained significant in the multivariate analysis after adjusting for age, gender, diabetes mellitus, hypertension, and BMI (β in model 1: 0.09 and 0.07 for hands ESC and feet ESC, respectively; Table 3). However, when the model was further adjusted for factors related to CKD, including hemoglobin, serum albumin, and UPCR, these associations became insignificant (model 2; Table 3). Similar results were observed in the subgroup of CKD patients with diabetes (β in univariate analysis: 0.18 and 0.15 for hands ESC and feet ESC, respectively; β in model 1: 0.15 and 0.13 for hands ESC and feet ESC, respectively; Table 4). However, in the subgroup of CKD patients without diabetes, the association of ESC with eGFR was only significant in the univariate analysis (β: 0.13 and 0.10 for hands ESC and feet ESC, respectively; Table 4).

In the multivariate linear regression analysis for all CKD participants, age, diabetes, and serum albumin showed significant associations with both hands ESC (β in model 2: −0.40, −4.89, and 7.62, respectively; Table 3) and feet ESC (β in model 2: −0.38, −7.27, and 8.80, respectively; Table 3). Additionally, BMI and hemoglobin were significantly associated with hands ESC (β in model 2: 0.41 and 1.46, respectively; Table 3). The outcomes of the multivariate logistic regression analysis for all CKD participants are presented in Supplementary Table S1.

For CKD patients with diabetes, the multivariate linear regression analysis revealed that hands ESC showed significant associations with age, BMI, hemoglobin, and serum albumin (β in model 2: −0.21, 0.47, 1.32, and 10.77, respectively; Table 4). On the other hand, feet ESC was only significantly associated with serum albumin (β in model 2: 12.68; Table 4). The outcomes of the multivariate logistic regression analysis for CKD patients with diabetes are presented in Supplementary Table S2.

For CKD patients without diabetes, the multivariate linear regression analysis showed a significant association between hands ESC and age, in addition to hemoglobin (β in model 2: −0.48 and 1.75, respectively; Table 4). Feet ESC exhibited a significant association with age and serum albumin (β in model 2: −0.49 and 6.88, respectively; Table 4). The outcomes of the multivariate logistic regression analysis for CKD patients without diabetes are presented in Supplementary Table S2.

### 3.4. The Association between Hands and Feet Sudoscan Score, Stratified by DM Status

**Figure 3:**
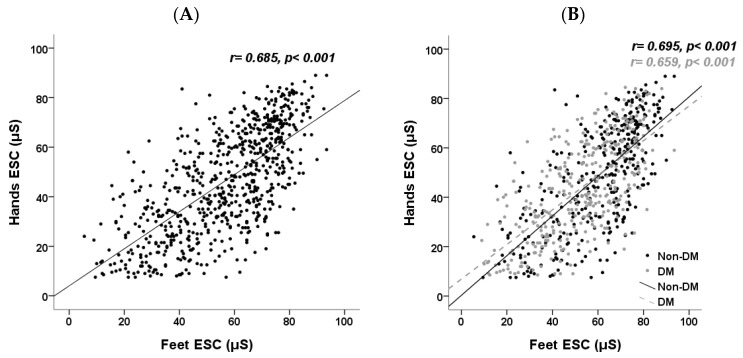
Scatter plots with linear regression illustrate the relationship between hands ESC and feet ESC in (**A**) all CKD participants and (**B**) CKD patients categorized by DM status. ESC, electrochemical skin conductance; CKD, chronic kidney disease; DM, diabetes mellitus.

The relationship between hands ESC and feet ESC was depicted in scatter plots and analyzed using Spearman’s correlation coefficient (Figure 3). There was a significant correlation between hands and feet ESC (r = 0.685, *p* < 0.001). This correlation remained significant regardless of DM status (Non-DM: r = 0.695, *p* < 0.001; DM: r = 0.659, *p* < 0.001; Figure 3).

### 3.5. The Relationship between Sudomotor Function and Clinical Neuropathy Scores

The association between hands and feet ESC and clinical neuropathy scores was obtained in 421 participants (CKD Stage 1–2/3/4–5: 102/187/123 patients), comprising 220 with diabetes and 201 without diabetes, using Spearman’s correlation analysis. As presented in Table 5, MNSI_Q (r = −0.20, *p* < 0.001; r = −0.17, *p* < 0.001), MNSI_P (r = −0.26, *p* < 0.001; r = −0.27, *p* < 0.001), and DN4 scores (r = −0.18, *p* < 0.001; r = −0.19, *p* < 0.001) all exhibited a significant negative correlation with hands and feet ESC.

## 4. Discussion

This study investigated the distribution of sudomotor function across stages of CKD. Both hands and feet ESC showed a significant decrease, and the prevalence of pathological hands and feet ESC increased with CKD progression, regardless of diabetes status. Diabetic CKD subjects had more impaired sudomotor function compared to non-diabetic CKD patients. Additionally, a correlation was observed between clinical neuropathy scores and sudomotor function. The small unmyelinated C fibers, similar in structure to the nerve fibers responsible for thermal perception, are also vulnerable to injury from metabolic derangements [17]. Sudomotor function reflects the activity of these small unmyelinated C fibers, allowing the assessment of peripheral sympathetic function through the measurement of sweat gland response using Sudoscan [17]. In diabetic patients, Sudoscan has been validated against other tools for neuropathy detection, including small-fiber neuropathy and autonomic dysfunction [12,22]. In a study by Casellini et al., Sudoscan demonstrated a sensitivity of 78% and a specificity of 92% for diagnosing diabetic peripheral neuropathy based on Toronto classification. Both hands and feet ESC correlated with the Neuropathy Impairment Score of Lower Legs and measures of autonomic function. Moreover, feet ESC showed a significant correlation with nerve conduction studies (peroneal nerve and sural nerve), vibration detection, and warm and cold perception in the big toe [12,17]. In the study by Selvarajah, both hands and feet ESC exhibited a significant correlation with the vibration detection threshold in the foot [23]. Additionally, Sudoscan has shown comparable diagnostic accuracy for intraepidermal nerve fiber density in diabetic distal symmetric polyneuropathy [10].

Uremic peripheral neuropathy is prevalent among CKD patients and typically affects large-diameter axons, resulting in demyelination and axonal degeneration. This neuropathy is characterized by symptoms such as numbness, weakness, and reduced reflexes, leading to physical dysfunction and decreased quality of life in both diabetic and non-diabetic patients [2,4,7]. A study involving 176 dialysis patients found that a pathological hands or feet ESC, measured by Sudoscan, was associated with a higher risk of intra-dialytic hypotension [18].

Studies have suggested that small-fiber involvement in uremic neuropathy is more prevalent than previously reported. Thermal sensation abnormalities have been observed in 30% of the CKD population, and these abnormalities do not correlate with large fiber dysfunction. This indicates that small fiber neuropathy can be considered a distinct form of uremic neuropathy [24]. In fact, the prevalence of small-fiber sensory neuropathy (67.5%) is higher compared to that of large-fiber neuropathies (37.5%) in patients with advanced CKD [25]. Furthermore, it has been widely accepted that uremic peripheral neuropathy is only observed in patients with advanced CKD [2,3]. However, recent studies have shown that neuropathy affects a significant proportion of patients with CKD stages 3 [7,26].

Previous studies examining the application of the Sudoscan score in CKD have mainly focused on diabetic patients and had a limited number of cases. For example, Freedman et al. demonstrated a significant association between average hands and feet ESC and eGFR in African Americans (but not in Caucasians) after adjusting for age, gender, BMI, and HbA1c in a cohort of 390 participants with diabetes [13]. The mean eGFR for African Americans and Caucasians in this study was 87.95 and 82.7 mL/min/1.73 m^2^, respectively, and the percentages of patients with GFR < 60 mL/min/1.73 m^2^ were only 9% and 16%, respectively. In another study of 2833 Hong Kong Chinese adults with diabetes, a low Sudoscan nephropathy score (calculated via the machine’s built-in algorithms) was significantly associated with low eGFR, even after adjusting for disease vintage, gender, BMI, blood pressure, HbA1c, cholesterol, and use of anti-hypertensive drugs [14]. In this study, the majority of participants did not have CKD (*n* = 2670), and only 163 patients had CKD, with a mean eGFR of 42.1 mL/min/1.73 m^2^ and a median urine albumin-to-creatinine ratio of 35.8 mg/mmol (0.32 g/g). In our study, we confirm the positive association between ESC and eGFR in both diabetic and non-diabetic patients with pre-dialysis CKD. Furthermore, after adjusting for CKD-associated factors, renal function itself was not found to be an independent predictor of hands and feet ESC.

In the present study, age, BMI, hemoglobin, and albumin were found to be significantly associated with hands or feet ESC in the fully adjusted model among diabetic patients. These findings are consistent with previous studies on peripheral neuropathy. Hypoalbuminemia and anemia have been reported to be associated with peripheral neuropathy in diabetic patients [27,28]. Furthermore, the association between albumin and diabetic neuropathy was found to be stronger in patients with a higher BMI (≥24 kg/m^2^) compared to those with a lower BMI (<24 kg/m^2^) [29]. In patients who initiated dialysis, the use of erythropoietin-stimulating agents significantly improved nerve conduction [30], and in pediatric patients with iron deficiency anemia, peripheral neuropathy was observed to reverse after iron treatment [31]. These findings suggest that malnutrition and anemia are likely universal factors contributing to peripheral neuropathy.

To the best of our knowledge, our study recruited the largest cohort for the measurement of sudomotor function across various stages of CKD. However, there are several limitations to consider. Firstly, we measured only clinical neuropathy scores in a subset of patients, and there is a lack of nerve conduction studies or other nerve function tests. Secondly, we did not rule out other potential causes of neuropathy, such as chronic inflammation, autoimmune disease, or chemotherapy agent use, which could potentially impact sudomotor function. Thirdly, relevant factors associated with uremic neuropathy, such as parathyroid hormone, thiamine, zinc, biotin, or β2-microglobulin, were not included in our study. Fourthly, sudomotor dysfunction reflects a combination of peripheral neuropathy and autonomic neuropathy, as indicated by the association with intradialytic hypotension demonstrated by Reach et al. [18]. We did not record symptoms related to autonomic dysfunction or assess autonomic function. Hence, we cannot definitively ascertain the influence of autonomic neuropathy on the study results. These aspects should be taken into consideration when designing future studies.

## 5. Conclusions

Pathological sudomotor function is present in all stages of non-dialysis CKD patients but is more common in stages 4–5. Furthermore, patients with diabetes exhibit more pronounced sudomotor dysfunction compared to non-diabetics. The correlation between clinical neuropathy scores and sudomotor function suggests that Sudoscan may predict peripheral neuropathy in patients with CKD. Although sudomotor dysfunction develops as renal diseases progress, eGFR is not identified as an independent risk factor. The factors contributing to uremic neuropathy require further exploration.

## Figures and Tables

**Table 1:** Clinical and biochemical characteristics of the study population.

Variables	Total (*n* = 700)	Stage 1–2 CKD (*n* = 143)	Stage 3 CKD (*n* = 303)	Stage 4–5 CKD (*n* = 254)	*p* for Trend
Basic demographic					
Age (years)	67 (59–76)	62 (50–69)	68 (61–78)	69 (61–78)	<0.001 *
Male, *n* (%)	404 (57.7)	78 (54.2)	196 (64.7)	130 (50.8)	0.201
Diabetes mellitus, *n* (%)	344 (49.1)	75 (52.1)	127 (41.9)	139 (54.3)	0.315
Hypertension, *n* (%)	546 (78.1)	94 (65.3)	228 (75.2)	224 (87.5)	<0.001 *
Glomerulonephritis, *n* (%)	236 (33.7)	45 (31.5)	95 (31.4)	96 (37.8)	0.141
Body mass index (kg/m^2^)	25 (23–28)	25 (24–29)	25 (23–28)	25 (22–28)	0.016 *
Laboratory values					
Hemoglobin (g/dL)	12.0 (10.5–13.8)	13.6 (12.4–14.8)	12.8 (11.3–14.3)	10.6 (9.4–11.5)	<0.001 *
Albumin (g/dL)	4.1 (3.9–4.3)	4.2 (4.0–4.5)	4.2 (4.0–4.3)	4.0 (3.8–4.2)	<0.001 *
BUN (mg/dL)	28 (20–45)	15 (12–19)	24 (20–30)	51 (39–69)	<0.001 *
Creatinine (mg/dL)	1.7 (1.2–2.8)	0.9 (0.7–1.1)	1.6 (1.4–1.8)	3.5 (2.6–5.3)	<0.001 *
eGFR (mL/min)	37.9 (21.5–54.6)	78.9 (66.6–96.5)	43.3 (35.7–49.6)	17.4 (9.3–22.5)	<0.001 *
UPCR (g/g)	0.40 (0.16–1.34)	0.22 (0.14–0.54)	0.25 (0.12–0.61)	1.24 (0.49–2.60)	<0.001 *
Total cholesterol (mg/dL)	151 (128–176)	155 (133–180)	152 (128–176)	144 (125–171)	0.096
HbA1c (%) ^a^	6.7 (6.1–7.5)	6.6 (6.1–7.6)	6.7 (6.1–7.6)	6.6 (5.9–7.5)	0.692
Calcium (mg/dL) ^b^	9.2 (8.9, 9.6)	9.3 (8.8, 9.6)	9.3 (9.0, 9.6)	9.0 (8.7, 9.4)	<0.001 *
Phosphorus (mg/dL) ^b^	3.6 (3.2–4.0)	3.5 (3.1–3.8)	3.4 (3.1–3.7)	3.9 (3.4–4.5)	<0.001 *
Hands					
ESC (μS)	46.5 (30.5–63.5)	54.0 (39.0–68.0)	45.5 (30.0–63.0)	41.8 (26.5–60.5)	<0.001 *
Pathological, *n* (%)	278 (39.7)	38 (26.6)	124 (40.9)	116 (45.7)	<0.001 *
Asymmetry (%)	8 (3–15)	5 (2–13)	8 (3–15)	9 (3–15)	0.008 *
Feet					
ESC (μS)	60.0 (43.0–72.0)	64.5 (53.5–74.0)	60.5 (43.0–72.5)	55.0 (39.0–69.8)	<0.001 *
Pathological, *n* (%)	237 (33.9)	31 (21.7)	103 (34.0)	103 (40.6)	<0.001 *
Asymmetry (%)	5 (2–10)	3 (1–7)	5 (2–10)	6 (2–13)	0.006 *
CAN score	33 (28–38)	32 (25–37)	34 (29–38)	33 (28–38)	0.004 *
Nephropathy score	46 (34–58)	55 (44–69)	44 (33–56)	44 (31–56)	<0.001 *

Data are presented as median (inter-quartile range) or number of patients (%). ^a^ The measurement was taken from 344 participants with DM. ^b^ The measurement was taken from 509 participants (stage 1–2: *n* = 66; stage 3: *n* = 239; stage 4–5: *n* = 204). CKD, chronic kidney disease; BUN, blood urea nitrogen; eGFR, estimated glomerular filtration rate; UPCR, urine protein to creatinine ratio; HbA1c, glycated hemoglobin; ESC, electrochemical skin conductance; CAN score, cardiac autonomic neuropathy score. A pathological ESC was defined by a hands ESC value of <40 µS or a feet ESC value of <50 µS. * *p* < 0.05.

**Table 2:** Spearman’s correlations between hands and feet ESC with clinical and biochemical characteristics.

	ESC (μS)			
	Hands		Feet	
Variables	*r*	*p*	*r*	*p*
Age (years)	−0.32	<0.001 *	−0.32	<0.001 *
Male, *n* (%)	0.04	0.254	0.03	0.465
Diabetes mellitus, *n* (%)	−0.14	<0.001 *	−0.23	<0.001 *
Hypertension, *n* (%)	−0.10	0.009 *	−0.09	0.013 *
Body mass index (kg/m^2^)	0.14	<0.001 *	0.06	0.091
Hemoglobin (g/dL)	0.28	<0.001 *	0.22	<0.001 *
Albumin (g/dL)	0.24	<0.001 *	0.24	<0.001 *
BUN (mg/dL)	−0.15	<0.001 *	−0.12	0.002 *
Creatinine (mg/dL)	−0.18	<0.001 *	−0.15	<0.001 *
eGFR (mL/min)	0.21	<0.001 *	0.18	<0.001 *
UPCR (mg/g)	−0.15	<0.001 *	−0.12	0.002 *
Total cholesterol (mg/dL)	0.06	0.121	0.08	0.044 *
HbA1c (%) ^a^	−0.04	0.518	−0.04	0.466
Calcium (mmol/L) ^b^	0.08	0.064	0.06	0.204
Phosphorus (mg/dL) ^b^	−0.05	0.228	−0.03	0.536

^a^ The measurement was taken from 344 participants with DM. ^b^ The measurement was taken from 509 participants (stage 1–2: *n* = 66; stage 3: *n* = 239; stage 4–5: *n* = 204). ESC, electrochemical skin conductance; BUN, blood urea nitrogen; eGFR, estimated glomerular filtration rate; UPCR, urine protein to creatinine ratio; HbA1c, glycated hemoglobin. * *p* < 0.05.

**Table 3:** Univariate and multivariate linear regression analysis of clinical factors associated with hands and feet ESC in all CKD participants.

Variables	Hands ESC (μS)			Feet ESC (μS)		
	β (95% CI)			β (95% CI)		
	Univariate	Multivariate		Univariate	Multivariate	
		Model 1	Model 2		Model 1	Model 2
Age (years)	−0.48 (−0.59, −0.37) *	−0.38 (−0.50, −0.27) *	−0.40 (−0.52, −0.28) *	−0.43 (−0.53, −0.33) *	−0.35 (−0.46, −0.25) *	−0.38 (−0.49, −0.27) *
DM, *n* (%)	−5.44 (−8.45, −2.42) *	−4.87 (−7.79, −1.95) *	−4.89 (−7.88, −1.89) *	−8.26 (−10.97, −5.55) *	−7.30 (−9.96, −4.64) *	−7.27 (−10.04, −4.49) *
BMI (kg/m^2^)	0.63 (0.30, 0.97) *	0.61 (0.29, 0.94) *	0.41 (0.07, 0.74) *	0.21 (−0.10, 0.52)	0.23 (−0.06, 0.53)	0.14 (−0.17, 0.45)
eGFR (mL/min)	0.16 (0.11, 0.21) *	0.09 (0.03, 0.14) *	−0.01 (−0.08, 0.06)	0.13 (0.08, 0.18) *	0.07 (0.02, 0.12) *	0.00 (−0.06, 0.07)
Hb (g/dL)	2.55 (1.90, 3.21) *	-	1.46 (0.61, 2.31) *	1.68 (1.07, 2.30) *	-	0.57 (−0.22, 1.36)
Albumin (g/dL)	7.99 (4.99, 11.00) *	-	7.62 (3.27, 11.97) *	6.91 (4.11, 9.70) *	-	8.80 (4.76, 12.84) *
UPCR (g/g)	−0.82 (−1.43, −0.21) *	-	−0.30 (−0.96, 0.35)	−0.83 (−1.39, −0.27) *	-	−0.26 (−0.87, 0.35)

Model 1 was adjusted for age, gender, diabetes mellitus, hypertension, body mass index and eGFR. Model 2 was further adjusted for hemoglobin, albumin and UPCR. ESC, electrochemical skin conductance; CKD, chronic kidney disease; CI, confidence interval; DM, diabetes mellitus; BMI, body mass index; eGFR, estimated glomerular filtration rate; Hb, hemoglobin; UPCR, urine protein to creatinine ratio. * *p* < 0.05.

**Table 4:** Univariate and multivariate linear regression analysis of clinical factors associated with hands and feet ESC in CKD patients stratified by DM status.

**DM (*n* = 344)**						
**Variables**	**Hands ESC (μS)**			**Feet ESC (μS)**		
	**β (95% CI)**			**β (95% CI)**		
	**Univariate**	**Multivariate**		**Univariate**	**Multivariate**	
		**Model 1**	**Model 2**		**Model 1**	**Model 2**
Age (years)	−0.31 (−0.49, −0.13) *	−0.17 (−0.36, 0.01)	−0.21 (−0.41, 0.00) *	−0.18 (−0.35, −0.01) *	−0.09 (−0.27, 0.09)	−0.12 (−0.32, 0.08)
BMI (kg/m^2^)	0.81 (0.37, 1.25) *	0.60 (0.16, 1.05) *	0.47 (0.01, 0.92) *	0.40 (−0.03, 0.82)	0.22 (−0.21, 0.65)	0.17 (−0.28, 0.62)
eGFR (mL/min)	0.18 (0.11, 0.25) *	0.15 (0.07, 0.22) *	−0.02 (−0.13, 0.09)	0.15 (0.08, 0.22) *	0.13 (0.06, 0.20) *	−0.02 (−0.12, 0.09)
Hb (g/dL)	2.22 (1.36, 3.08) *	-	1.32 (0.10, 2.54) *	1.62 (0.78, 2.46) *	-	0.80 (−0.40, 2.00)
Albumin (g/dL)	14.37 (9.33, 19.41) *	-	10.77 (4.64, 16.90) *	14.51 (9.63, 19.39) *	-	12.68 (6.65, 18.71) *
UPCR (g/g)	−0.76 (−1.41, −0.11) *	-	−0.18 (−0.93, 0.57)	−0.81 (−1.43, −0.19) *	-	−0.13 (−0.87, 0.61)
**Non-DM (*n* = 356)**						
**Variables**	**Hands ESC (μS)**			**Feet ESC (μS)**		
	**β (95% CI)**			**β (95% CI)**		
	**Univariate**	**Multivariate**		**Univariate**	**Multivariate**	
		**Model 1**	**Model 2**		**Model 1**	**Model 2**
Age (years)	−0.55 (−0.69, −0.41) *	−0.51 (−0.66, −0.37) *	−0.48 (−0.63, −0.33) *	−0.53 (−0.65, −0.42) *	−0.52 (−0.64, −0.40) *	−0.49 (−0.62, −0.36) *
BMI (kg/m^2^)	0.71 (0.19, 1.23) *	0.68 (0.19, 1.17) *	0.42 (−0.08, 0.93)	0.35 (−0.09, 0.80)	0.30 (−0.11, 0.72)	0.21 (−0.23, 0.65)
eGFR (mL/min)	0.13 (0.05, 0.21) *	0.03 (−0.05, 0.11)	−0.02 (−0.11, 0.08)	0.10 (0.04, 0.17) *	0.01 (−0.06, 0.08)	0.01 (−0.07, 0.09)
Hb (g/dL)	2.86 (1.89, 3.83) *	-	1.75 (0.54, 2.97) *	1.67 (0.81, 2.53) *	-	0.57 (−0.48, 1.63)
Albumin (g/dL)	4.75 (0.98, 8.51) *	-	5.76 (−0.70, 12.22)	2.92 (−0.36, 6.20)	-	6.88 (1.27, 12.48) *
UPCR (g/g)	−0.27 (−1.74, 1.20)	-	0.25 (−1.29, 1.78)	0.28 (−0.97, 1.54)	-	0.55 (−0.78, 1.88)

Model 1 was adjusted for age, gender, diabetes mellitus, hypertension, body mass index and eGFR. Model 2 was further adjusted for hemoglobin, albumin and UPCR.ESC, electrochemical skin conductance; CKD, chronic kidney disease; DM, diabetes mellitus; CI, confidence interval; BMI, body mass index; eGFR, estimated glomerular filtration rate; Hb, hemoglobin; UPCR, urine protein to creatinine ratio. * *p* < 0.05.

**Table 5:** Spearman’s correlations between hands and feet ESC with clinical neuropathy scores in 421 patients.

	ESC (μS)			
	Hands		Feet	
Variables	*r*	*p*	*r*	*p*
MNSI_Q (score)	−0.20	<0.001 *	−0.19	<0.001 *
MNSI_P (score)	−0.26	<0.001 *	−0.30	<0.001 *
DN4 (score)	−0.18	<0.001 *	−0.22	<0.001 *

ESC, electrochemical skin conductance; MNSI_Q, Michigan Neuropathy Screening Instrument questionnaire; MNSI_P, Michigan Neuropathy Screening Instrument physical exam; DN4, Douleur Neuropathique en 4 questionnaire. * *p* < 0.05.

## Data Availability

The corresponding author can provide the supporting data for this study upon reasonable request.

## Supplementary Materials

The following supporting information can be downloaded at: https://www.mdpi.com/article/10.3390/jcm13010187/s1, Table S1: Odds ratios (95% CI) according to univariate and multivariate logistic regression analyses of risk factors associated with pathological hands and feet ESC; Table S2: Odds ratios (95% CI) according to univariate and multivariate logistic regression analyses of risk factors associated with pathological hands and feet ESC in DM and non-DM patients.
