# Supplementary material for: Electrochemical Skin Conductance by Sudoscan in Non-Dialysis Chronic Kidney Disease Patients
Source: J Clin Med. 2023 Dec 28;13(1):187. doi: 10.3390/jcm13010187 (PMC10779764; doi:10.3390/jcm13010187)
Supplement: Supplementary file 1 [file jcm-13-00187-s001.zip › jcm-2759484-supplementary.pdf]

Supplementary Table S1. Odds ratios (95% CI) according to univariate and multivariate logistic regression analyses of risk factors associated with pathological hands and feet ESC

Variables	Pathological hands ESC			Pathological feet ESC		
	Univariate	Multivariate		Univariate	Multivariate	
		Model 1	Model 2		Model 1	Model 2
Age (years)	1.05 (1.03, 1.06)*	1.04 (1.03, 1.06)*	1.05 (1.03, 1.06)*	1.05 (1.03, 1.06)*	1.04 (1.03, 1.06)*	1.05 (1.03, 1.07)*
DM, n (%)	1.52 (1.12, 2.06)*	1.56 (1.12, 2.17)*	1.53 (1.07, 2.18)*	2.21 (1.60, 3.04)*	2.21 (1.57, 3.13)*	2.38 (1.64, 3.46)*
BMI (kg/m^2)	0.94 (0.91, 0.98)*	0.94 (0.91, 0.98)*	0.97 (0.93, 1.01)	0.97 (0.94, 1.01)	0.97 (0.93, 1.01)	0.99 (0.95, 1.03)
CKD stages						
CKD stage 1-2	1.00 (reference)	1.00 (reference)	1.00 (reference)	1.00 (reference)	1.00 (reference)	1.00 (reference)
CKD stage 3	1.91 (1.24, 2.96)*	1.44 (0.90, 2.29)	1.06 (0.62, 1.82)	1.86 (1.17, 2.96)*	1.48 (0.90, 2.44)	0.93 (0.53, 1.63)
CKD stage 4-5	2.32 (1.49, 3.63)*	1.62 (1.00, 2.61)*	0.70 (0.37, 1.30)	2.46 (1.54, 3.94)*	1.75 (1.05, 2.90)	0.61 (0.32, 1.18)
Hb (g/dL)	0.81 (0.75, 0.87)*	-	0.85 (0.76, 0.94)*	0.83 (0.77, 0.90)*	-	0.87 (0.78, 0.98)*
Albumin (g/dL)	0.31 (0.20, 0.48)*	-	0.50 (0.29, 0.85)*	0.29 (0.19, 0.45)*	-	0.41 (0.24, 0.71)*
UPCR (g/g)	1.10 (1.02, 1.18)*	-	1.08 (1.00, 1.17)	1.12 (1.04, 1.20)*	-	1.07 (0.99, 1.16)

A pathological ESC was defined by a hands ESC value of < 40 µS or a feet ESC value of < 50 µS.

Model 1 was adjusted for age, gender, diabetes mellitus, hypertension, body mass index and CKD stages. Model 2 was further adjusted for hemoglobin, albumin and UPCR.

CI, confidence interval; ESC, electrochemical skin conductance; DM, diabetes mellitus; BMI, body mass index; CKD, chronic kidney disease; Hb, hemoglobin; UPCR, urine protein to creatinine ratio.

*$p < 0.05$.

Supplementary Table S2. Odds ratios (95% CI) according to univariate and multivariate logistic regression analyses of risk factors associated with pathological hands and feet ESC in DM and non-DM patients

**DM (n= 344)**						
Variables	Pathological hands ESC			Pathological feet ESC		
	Univariate	Multivariate		Univariate	Multivariate	
		Model 1	Model 2		Model 1	Model 2
Age (years)	1.03 (1.01, 1.05)*	1.02 (1.00, 1.04)	1.02 (1.00, 1.05)	1.02 (1.00, 1.04)	1.01 (0.99, 1.03)	1.01 (0.99, 1.04)
BMI (kg/m^2)	0.94 (0.89, 0.99)*	0.95 (0.90, 1.00)	0.97 (0.92, 1.03)	0.95 (0.90, 1.00)*	0.96 (0.91, 1.01)	0.97 (0.91, 1.02)
CKD stages						
CKD stage 1-2	1.00 (reference)	1.00 (reference)	1.00 (reference)	1.00 (reference)	1.00 (reference)	1.00 (reference)
CKD stage 3	1.58 (0.87, 2.87)	1.38 (0.75, 2.56)	0.73 (0.35, 1.55)	2.04 (1.10, 3.80)*	1.91 (1.01, 3.59)*	0.85 (0.40, 1.78)
CKD stage 4-5	2.57 (1.43, 4.63)*	2.14 (1.16, 3.95)*	0.61 (0.26, 1.47)	3.05 (1.66, 5.61)*	2.70 (1.44, 5.08)*	0.60 (0.25, 1.44)
Hb (g/dL)	0.81 (0.73, 0.89)*	-	0.84 (0.72, 0.98)*	0.83 (0.75, 0.92)*	-	0.89 (0.76, 1.03)
Albumin (g/dL)	0.20 (0.10, 0.39)*	-	0.32 (0.14, 0.70)*	0.18 (0.09, 0.36)*	-	0.22 (0.10, 0.51)*
UPCR (g/g)	1.12 (1.02, 1.23)*	-	1.06 (0.96, 1.16)	1.16 (1.05, 1.28)*	-	1.04 (0.95, 1.15)
**Non-DM (n= 356)**						
Variables	Pathological hands ESC			Pathological feet ESC		
	Univariate	Multivariate		Univariate	Multivariate	
		Model 1	Model 2		Model 1	Model 2
Age (years)	1.07 (1.04, 1.09)*	1.06 (1.04, 1.09)*	1.06 (1.04, 1.09)*	1.09 (1.06, 1.12)*	1.09 (1.06, 1.12)*	1.08 (1.05, 1.12)*
BMI (kg/m^2)	0.93 (0.88, 0.98)*	0.92 (0.86, 0.98)*	0.94 (0.88, 1.01)	0.97 (0.92, 1.03)	0.97 (0.90, 1.04)	0.99 (0.92, 1.07)
CKD stages						
CKD stage 1-2	1.00 (reference)	1.00 (reference)	1.00 (reference)	1.00 (reference)	1.00 (reference)	1.00 (reference)
CKD stage 3	2.52 (1.30, 4.89)*	1.33 (0.64, 2.76)	1.29 (0.56, 2.95)	1.98 (0.96, 4.09)	0.86 (0.38, 1.96)	0.77 (0.31, 1.93)
CKD stage 4-5	2.05 (1.01, 4.13)*	1.05 (0.48, 2.31)	0.67 (0.25, 1.78)	1.82 (0.84, 3.92)	0.76 (0.32, 1.84)	0.46 (0.16, 1.37)
Hb (g/dL)	0.82 (0.73, 0.91)*	-	0.85 (0.73, 1.00)*	0.83 (0.74, 0.93)*	-	0.83 (0.70, 0.99)*
Albumin (g/dL)	0.48 (0.26, 0.87)*	-	0.66 (0.29, 1.50)	0.47 (0.25, 0.89)*	-	0.65 (0.27, 1.60)
UPCR (g/g)	1.00 (0.86, 1.15)	-	1.04 (0.86, 1.25)	0.92 (0.77, 1.11)	-	0.96 (0.76, 1.20)

A pathological ESC was defined by a hands ESC value of < 40 µS or a feet ESC value of < 50 µS.

Model 1 was adjusted for age, gender, diabetes mellitus, hypertension, body mass index and CKD stages. Model 2 was further adjusted for hemoglobin, albumin and UPCR.

CI, confidence interval; ESC, electrochemical skin conductance; DM, diabetes mellitus; BMI, body mass index; CKD, chronic kidney disease; Hb, hemoglobin; UPCR, urine protein to creatinine ratio.

*$p < 0.05$.
